# An Observational Study of Circulating Tumor Cells and ^18^F-FDG PET Uptake in Patients with Treatment-Naive Non-Small Cell Lung Cancer

**DOI:** 10.1371/journal.pone.0067733

**Published:** 2013-07-05

**Authors:** Viswam S. Nair, Khun Visith Keu, Madelyn S. Luttgen, Anand Kolatkar, Minal Vasanawala, Ware Kuschner, Kelly Bethel, Andrei H. Iagaru, Carl Hoh, Joseph B. Shrager, Billy W. Loo, Lyudmila Bazhenova, Jorge Nieva, Sanjiv S. Gambhir, Peter Kuhn

**Affiliations:** 1 Division of Pulmonary & Critical Care Medicine, Stanford University School of Medicine, Stanford, California, United States of America; 2 Division of Nuclear Medicine and Molecular Imaging, Stanford University School of Medicine, Stanford, California, United States of America; 3 Department of Cell Biology, The Scripps Research Institute, La Jolla, California, United States of America; 4 Division of Nuclear Medicine, Veterans Affairs Palo Alto Health Care System, Palo Alto, California, United States of America; 5 Pulmonary & Critical Care Medicine Section, Veterans Affairs Palo Alto Health Care System, Palo Alto, California, United States of America; 6 Department of Pathology, Scripps Clinic, La Jolla, California, United States of America; 7 Department of Radiology, University of California San Diego Medical Center, La Jolla, California, United States of America; 8 Division of Thoracic Surgery, Veterans Affairs Palo Alto Health Care System, Palo Alto, California, United States of America; 9 Division of Thoracic Surgery, Stanford University School of Medicine, Stanford, California, United States of America; 10 Department of Radiation Oncology, Stanford University School of Medicine, Stanford, California, United States of America; 11 Moores Cancer Center, University of California San Diego Medical Center, La Jolla, California, United States of America; 12 The Department of Hematology and Oncology, Billings Clinic, Billings, Montana, United States of America; 13 Department of Radiology, Stanford University School of Medicine, Stanford, California, United States of America; 14 The Molecular Imaging Program at Stanford, Stanford University School of Medicine, Stanford, California, United States of America; Univesity of Texas Southwestern Medical Center at Dallas, United States of America

## Abstract

**Introduction:**

We investigated the relationship of circulating tumor cells (CTCs) in non-small cell lung cancer (NSCLC) with tumor glucose metabolism as defined by ^18^F-fluorodeoxyglucose (FDG) uptake since both have been associated with patient prognosis.

**Materials & Methods:**

We performed a retrospective screen of patients at four medical centers who underwent FDG PET-CT imaging and phlebotomy prior to a therapeutic intervention for NSCLC. We used an Epithelial Cell Adhesion Molecule (EpCAM) independent fluid biopsy based on cell morphology for CTC detection and enumeration (defined here as High Definition CTCs or “HD-CTCs”). We then correlated HD-CTCs with quantitative FDG uptake image data calibrated across centers in a cross-sectional analysis.

**Results:**

We assessed seventy-one NSCLC patients whose median tumor size was 2.8 cm (interquartile range, IQR, 2.0–3.6) and median maximum standardized uptake value (SUV_max_) was 7.2 (IQR 3.7–15.5). More than 2 HD-CTCs were detected in 63% of patients, whether across all stages (45 of 71) or in stage I disease (27 of 43). HD-CTCs were weakly correlated with partial volume corrected tumor SUV_max_ (r = 0.27, p-value = 0.03) and not correlated with tumor diameter (r = 0.07; p-value = 0.60). For a given partial volume corrected SUV_max_ or tumor diameter there was a wide range of detected HD-CTCs in circulation for both early and late stage disease.

**Conclusions:**

CTCs are detected frequently in early-stage NSCLC using a non-EpCAM mediated approach with a wide range noted for a given level of FDG uptake or tumor size. Integrating potentially complementary biomarkers like these with traditional patient data may eventually enhance our understanding of clinical, *in vivo* tumor biology in the early stages of this deadly disease.

## Introduction

Two of the most active areas of inquiry in cancer research today are focused on putative circulating tumor cells (CTCs) that are released from the parent tumor into blood [1] and molecular imaging agents that can define tumor biology *in vivo* [2]. This is driven in part by the belief that both of these technologies are potentially robust, cost effective, and readily translatable to the clinic with a minimum risk to the patient.

^18^F-fluoro-2-deoxy-*D*-glucose (FDG) PET is currently the only widely used molecular imaging agent clinically, and it capitalizes on glucose metabolism to capture a snapshot of unperturbed tumor biology at diagnosis [3], [4]. While many studies have assessed [5] whether the intensity of FDG uptake may relate to a tumor’s metastatic potential via the Warburg Effect and deranged cellular bioenergetics [6]–[9], the mechanism for this association still remains poorly understood.

Current theories for how the “seed and soil” mechanism of tumor metastasis occurs posit that CTCs must first undergo an epithelial-to-mesenchymal transition (EMT) for release followed by a mesenchymal-to-epithelial (MET) transition for metastatic deposition in an adequate environment [10]–[13]. Since tumor glucose metabolism is driven by the Warburg Effect, during which aberrant aerobic glycolysis becomes evolutionarily advantageous [14], the initiating events of metastatic propagation may in part relate to more rapidly dividing tumors that have increased FDG uptake on PET [15].

How CTCs associate with tumor glucose metabolism remains largely unexplored clinically. To investigate this question, we report on the correlation of circulating tumor cells using a non-EpCAM based CTC assay with standardized, semi quantitative, tumor FDG uptake metrics in patients undergoing evaluation for treatment-naïve non-small cell lung cancer (NSCLC).

### Materials and Methods

### Study Design

This was a multi-center, cross-sectional analysis of existing data from ongoing observational studies. Data were obtained retrospectively from patients with NSCLC of all stages (American Joint Committee on Cancer, 7th edition) [16] that underwent FDG PET-CT imaging and CTC analysis from a peripheral blood draw between October 2009 and May 2012. We included those patients with NSCLC that had FDG PET-CT images acquired along with a CTC sample within 90 days and prior to a surgical, medical or combination treatment. Subjects who underwent a biopsy prior to enrollment were also allowed to participate.

**Figure 1:**
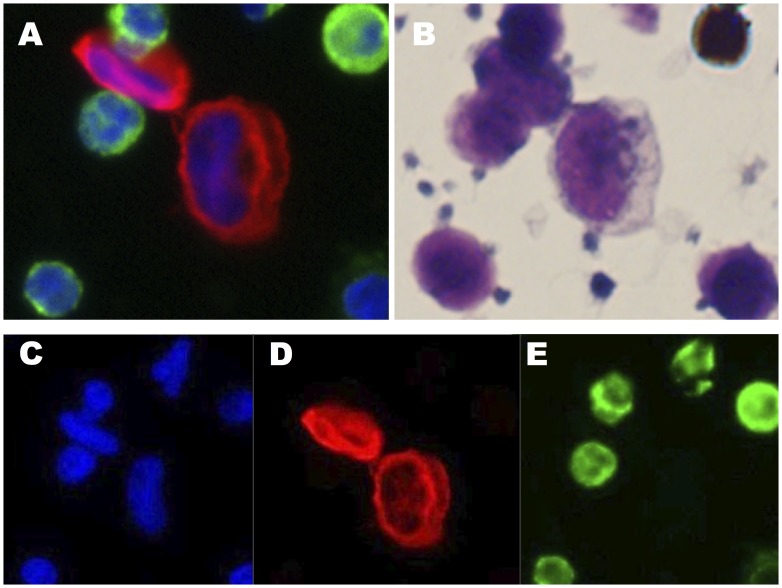
Detecting Putative DAPI(+), CK(+), CD45(-) HD-CTCs by Fluid Biopsy. A representative image of High Definition Circulating Tumor Cells (HD-CTCs) from a Stanford patient with stage I non-small cell lung cancer shown in composite immunofluorescence (A) and by Wright-Giemsa brightfield microscopy (B). HD-CTCs are characterized as 4′,6-diamidino-2-phenylindole (DAPI) positive with a nucleus that is larger than surrounding white blood cells (Blue, C), cytokeratin (CK) positive (Red, D) and CD45 leukocyte marker negative (Green, E).

Patients were enrolled consecutively at four sites: Stanford University Medical Center (SUMC); The Veterans Affairs Palo Alto Health Care System (VAPAHCS); The University of California San Diego Moores Cancer Center (UCSD); and the Billings Clinic (Billings) (Supplementary File 1, S Figure 1). Patients at SUMC and VAPAHCS were enrolled at the time of FDG PET-CT as part of a formal early-detection study examining circulating biomarkers and imaging, and patients at UCSD and Billings with any stage of disease were eligible if they met the inclusion criteria. Phlebotomy was performed using standard techniques and samples were processed at The Scripps Research Institute (TSRI) within 48 hours of phlebotomy (median time = 23 hours) [17]. Medical charts were reviewed to extract patient demographic, clinical, imaging and treatment information by the collaborating research team at each respective site. Stanford University, Billings Clinic and Scripps Research Institute Institutional Review Boards (IRBs) approved all work presented in this study at their respective sites. Fully informed, written patient consent was obtained prior to enrollment after review of study protocol documents. HD-CTC results for nine patients included for this CTC–imaging correlation study have previously been published [18].

### Circulating Tumor Cell Analysis

We used a non-EpCAM based, immunofluorescent, morphologic approach to quantify CTCs as described previously (Figure 1) [17]–[20]. CTCs were identified by immunofluorescence (a panel of cytokeratins, DAPI, CD45) with automated morphometric analysis followed by manual validation by a pathologist-trained technician (MSL). The technologist, microscopes and automated imaging system were constant throughout the study. We summarize the methods [17] here for completeness.

Six to 10 mL of whole blood collected into Cell-Free DNA BCT™ (Streck, Omaha, NE) was subjected to red blood cell lysis at room temperature, centrifuged and the resulting cellular pellet was re-suspended and attached as a monolayer to custom designed, glass slides. Four slides were processed to ensure adequate sample for analysis, usually representing 1–2 mL of whole blood. Prepared samples were stored at −80°C until proceeding to the staining protocol and analysis. Thawed slides were fixed, permeabilized and then incubated with a monoclonal anti-cytokeratin (epithelial cell marker) antibody that targets human cytokeratins (CK) 1, 4, 5, 6, 8, 10, 13, 18 and 19 (Sigma-Aldrich, St. Louis, MO); an AlexaFluor® 555 conjugated goat anti-mouse secondary antibody (Life Technologies, Carlsbad, CA); a monoclonal antibody targeting CD45 directly conjugated to a AlexaFluor® 647 dye (AbD Serotec, Oxford, UK); and a nuclear counterstain of 0.5 g/ml 4,6-diamidino-2-phenylindole (DAPI) (Life Technologies, Carlsbad, CA).

All four slides, that together comprised one “test,” were scanned in their entirety by an automated fluorescent microscope. Candidate cellular events were manually classified as *High Definition CTCs (HD-CTCs*) if they were CK positive, CD45 negative, contained an intact DAPI positive nucleus without identifiable apoptotic changes or a disrupted appearance, and were morphologically distinct from surrounding white blood cells (WBCs). HD-CTC enumeration was determined from the four-slide set with the goal of analyzing a total plated blood volume containing 1 x 10^7^ nucleated cells per test. WBC counts of whole blood were determined automatically (WBC system, HemoCue®, Cypress, CA) and the number of nucleated cells detected by the assay per slide (via DAPI and CD45 staining) was used to calculate the equivalent amount of blood analyzed per slide. This fluid biopsy platform also avoids discarding other abnormal cells with some HD-CTC characteristics that do not fully meet inclusion criteria (such as apoptotic bodies) and digitally catalogs them for subsequent analysis.

HD-CTC clusters for this study were identified as described previously [21], and were enumerated from spatial groupings and then characterized as total number of clusters. Clusters were defined as at least two HD-CTC cells with cytoplasm in contact with each other upon visual inspection during cell counting. We analyzed HD-CTCs on a continuous number scale standardized per 10 million WBCs (referred to as HD-CTC/10M WBC) and total HD-CTC clusters per sample. We also report on HD-CTCs standardized per blood volume as HD-CTC/mL for comparison to other existing platforms and for ease of interpretation. To calibrate the HD-CTC test, samples were analyzed for other cancers of the lung (non-NSCLC) and for documented benign nodules of the lung by biopsy, surgery or clinical follow-up. Importantly, analysis of all samples (ML) was performed blinded to diagnosis to remove any possible interpretation bias.

### HD-CTC Assay Reproducibility

The HD-CTC assay was technically validated with cell line spiking experiments to reach an R^2^ = 0.9997 on linearity testing as previously reported [17]. These experiments were performed using SKBR3 cell lines and 0 to 3 x 10^2^ cells per mL of normal donor control blood. The coefficient of variation (CV) for this assay is 16% and inter-processor correlation is R^2^ = 0.979. Sample preparation process adheres to standard operating procedures for patient samples through a bar-coded system for all consumables and instrumentation. All off-the-shelf instrumentation is calibrated according to the manufacturer’s recommendation and all custom instrumentation is calibrated according to the technical validation protocols established during commissioning.

### FDG PET-CT Acquisition

In general, FDG PET-CT acquisition was performed for the fasting patient (minimum six hours) after an injection of 444–555 MBq ^18^F-FDG, evaluation of the patient’s glucose level, and a tracer uptake period of 60–90 minutes prior to imaging. Ordered subset expectation maximization (OSEM) reconstruction with CT attenuated-correction was performed on PET data at all centers and detailed imaging protocols for each center are available in Supplementary File 1, Table S1. Semi-quantitative values (maximum standardized uptake value, SUV_max_) were extracted from FDG PET-CT images using a region of interest drawn over the tumor by the interpreting physician for further analysis per each institution’s clinical protocols. For cases where SUV_max_ was not reported at the time of dictation, available images were reviewed by the collaborating researcher at each institution (KVK at SUMC, MV at PAVAHCS, CH at UCSD and JN at Billings) to extract the value.

### Phantom Protocol

Partial volume correction (PVC) [22] was applied to SUV_max_ using data acquired from an anthropomorphic thoracic phantom that was scanned at each participating site (Supplementary File 1, Figure S2). Briefly, a phantom consisting of a mock mediastinal pool, two lungs and “tumor” spheres ranging from 0.4 cm to 3.1 cm with known and identical FDG concentrations mimicking tumor ranges in human, was scanned at each participating center using its clinical protocol. Acquired images were reconstructed using the participating center’s algorithm and then a recovery coefficient (RC) was generated according to previous methods [22]. This RC curve was used to correct for partial volume effects. We denote the corrected features in this study with a “PVC” subscript for distinction (i.e., PVC SUV_max_ is denoted as SUV_maxPVC_). Lastly, we used this curve to determine how well scanners were calibrated across centers (Supplementary File 1 Figure S3).

### FDG PET-CT Uptake Features

**Figure 2:**
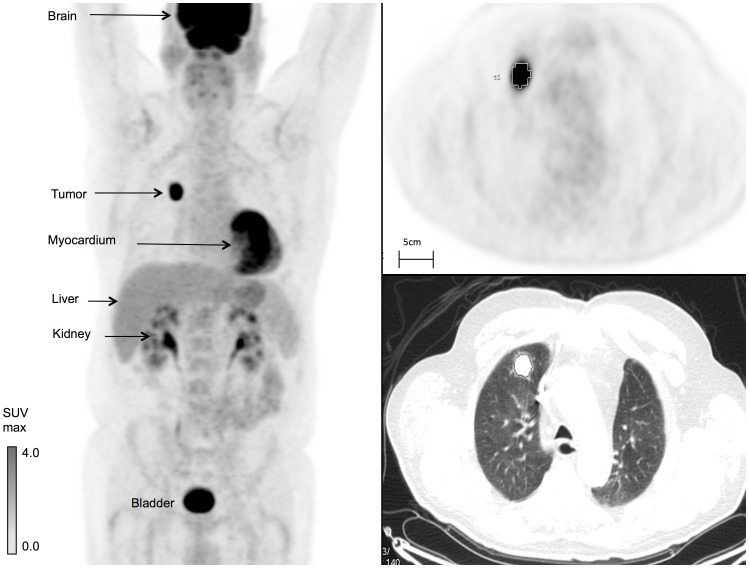
Non-small Cell Lung Cancer FDG PET-CT Imaging Features. A three dimensional, maximum intensity projection, whole body ^18^F-FDG PET-CT (left). Physiologic uptake is seen in the brain, heart and liver with excretion through the renal pelvis and bladder. This tumor showed an intense FDG uptake with SUV_max_ of 19, SUV_mean_ of 9.6, and TLG of 65.6 using a 50% SUV_max_ threshold (upper right). On CT, the lesion volume was estimated at 6.0 cm^3^ with a maximum diameter of 22 mm (lower right).

FDG uptake features were extracted using a three-dimensional region of interest (ROI) over the primary tumor on a GE Healthcare AW Workstation, v4.5 at SUMC with the PET VCAR™ implementation (Figure 2). PET-VCAR™ is a software platform provided by GE Healthcare with feature annotations available as an add-on to GE workstations. [23] We re-extracted SUV_max_ from the raw Digital Imaging in Communication of Medicine (DICOM) files for each institution to verify clinical interpretation of SUV_max_ at each site and to ensure reproducibility. Tumor SUV_maxPVC_ was calculated after extracting background FDG lung uptake and using RC phantom data (Supplementary File 1, SMethods and Table S2).

### FDG PET-CT Volumetric Analysis

Since we were interested in determining how Total Lesion Glycolysis (TLG, defined here as SUV_mean_ x metabolic tumor volume) [24] correlated with HD-CTCs when compared to anatomic tumor volume on the CT portion of the FDG PET-CT study, we used PET-VCAR™ to segment and calculate volumes from PET and CT images. For PET images, this threshold had a default setting of 50%, and it represented the signal drop-off that bounded the region-growing algorithm to define the FDG uptake. For cases where this segmentation did not accurately represent the tumor, a threshold was manually optimized to fully capture the FDG activity using the co-registered CT image. Anatomic tumor diameter and volume were captured from the CT image by visual inspection. Of note, the interpreting physician (KVK) was blinded to HD-CTC results.

## Statistical Analysis

Descriptive statistics for clinical, imaging and pathologic variables were determined using the median and interquartile range (IQR) or number with percent as appropriate. Differences across centers were assessed by ANOVA for continuous variables using Tukey’s test, a Chi-squared and Fisher’s exact test for categorical variables as appropriate, and a Kruskal-Wallis test for ordinal variables. Normality for variables included in modeling was formally tested using the Kolmogorov-Smirnov method. HD-CTC counts were correlated with FDG uptake using a Spearman rank test to account for the non-parametric distribution of the underlying variables. Log-normalized imaging and HD-CTC metrics were also compared using Spearman rank and Kendall’s Tau correlations to assess consistency. For additional analyses, we defined a FDG avid tumor as one with a SUV_max_ ≥2.5, since this is a clinically relevant distinction [25], and non-metastatic tumors as any T stage tumor without accompanying satellite lesions, nodal or distant metastasis, according to the most recent AJCC 7.0 guidelines.

Statistical analyses were performed using Excel™ (Excel for Mac, 2010, Seattle, WA) and SAS Enterprise Guide™ (v4.3, Cary, NC).

## Results

Of 153 patients examined across the four sites involved in this study (Supplementary File 1, Figure S1), seventy-one patients were ultimately eligible for analysis, and 62 of these patients had raw imaging (DICOM) files available for more detailed image feature extraction (Table 1). The median age for the cohort was 71 years (range 44–96 years), 59% were male gender, and the majority of patients were white (73%). Forty-three patients had stage I disease, 5 had stage II, and 23 had stage III-IV NSCLC, with the median primary tumor diameter being 2.8 cm (IQR 2.0–3.7 cm).

**Table 1:** Clinical, CTC and FDG PET-CT Patient Characteristics by Center.

Variable*		All (n = 71)	SUMC (n = 24)	UCSD (n = 19)	PAVAHCS (n = 17)	Billings (n = 11)
**CLINICAL DATA**	Age (years)	71 (65–79)	75 (66–81)	69 (64–78)	67 (66–75)	73 (61–82)
	Male gender†	42 (59)	14 (58)	7 (37)	15 (88)	6 (55)
	Ethnicity†					
	White	52 (73)	16 (67)	13 (68)	12 (71)	11 (100)
	Asian	5 (7)	5 (21)	0 (0)	0 (0)	0 (0)
	Black	5 (7)	0 (0)	2 (11)	3 (18)	0 (0)
	Other/unknown	9 (13)	3 (12)	4 (21)	2 (12)	0 (0)
	Tumor Diameter (mm)† §	28 (20–37)	26 (19–32)	37 (24–73)	22 (17–29)	36 (29–70)
	Tumor Volume (cm^3^)† §	7.6 (2.7–14.8)	6.1 (2.6–9.2)	20.3 (6.4–146)	4.0 (2.6–9.0)	14.5 (9.6–98.0)
	Tumor Histology					
	Adenocarcinoma	44 (62)	18 (75)	13 (68)	9 (53)	4 (36)
	Squamous	14 (20)	5 (21)	3 (16)	3 (18)	3 (27)
	Other NSCLC	13 (18)	1 (4)	3 (16)	5 (29)	4 (36)
	Stage (AJCC 7^th^ ed.)†					
	I	43 (61)	20 (83)	9 (47)	11 (65)	3 (27)
	II	5 (7)	2 (8)	2 (11)	0 (0)	1 (9)
	III	17 (24)	1 (4)	8(42)	2 (12)	6 (55)
	IV	6 (8)	1 (4)	0 (0)	4 (24)	1 (9)
**CTC DATA**	Time to processing (hrs)	23.1 (22.0–24.0)	23.3 (22.5–23.9)	23.5 (22.0–24.0)	22.8 (20.3–23.8)	23.0 (22.2–25.2)
	mL/test	1.23 (0.95–1.55)	1.38 (1.09–1.86)	1.23 (0.76–1.38)	1.20 (0.91–1.51)	1.14 (0.95–1.73)
	CTC/mL	3.4 (0.6–29.6)	4.1 (0.5–18.6)	2.4 (1.3–35.4)	1.8 (0.6–18.5)	41.2 (0.4–69.1)
	CTC/10 M WBC	5.4 (1.0–24.5)	6.6 (1.0–22.7)	4.3 (1.1–48.6)	2.5 (1.0–24.8)	36.8 (1.4–107)
	Total Clusters†	0 (0–5)	0 (0–3)	0 (0–6)	0 (0–2)	0 (0–20)
	Clusters Present (% yes)	33 (46)	11 (46)	8 (42)	7 (41)	7 (64)
	Cells in Clusters (%)	31.6 (0–46.2)	41.0 (0–56.5)	20.0 (0–47.9)	28.6 (0–44.4)	11.8 (0–41.7)
**FDG PET DATA**	Phlebotomy to PET (days)¶	108 (−24 to 84)	29 (−1 to 28)	108 (−24 to 84)	62 (−21 to 41)	12 (6 to 18)
	Injected Tracer Dose (MBq)†	525 (466–599)	455 (407–477)	651 (588–699)	551 (503–599)	537 (525–570)
	Time To Scan (min)†?	62 (59–79)	60 (55–70)	65 (58–85)	62 (58–69)	89 (79–94)
	Glucose (mg/dL)	101 (96–112)	104 (95–112)	97 (91–110)	99 (97–118)	103 (96–110)
	SUV_max_ † § ^≈^	7.2 (3.7–15.5)	5.1 (2.2–9.4)	7.9 (5.1–17.7)	5.6 (2.2–15.5)	17.5 (12.4–25.5)
	SUV_maxPVC_ † §	8.8 (4.5–16.8)	8.0 (4.2–10.4)	13.8 (3.2–18.9)	5.7 (4.2–16.8)	17.5 (16.0–25.5)
	Total Lesion Glycolysis§	26.5 (7.9–95.5)	15.6 (7.0–46.6)	83.7 (27.5–218)	21.0 (3.5–26.6)	117.0 (28.9–1,061)
	DICOM Files Available	62 (87)	24 (100)	11 (58)	17 (100)	10 (91)

SUMC = Stanford University Medical Center; UCSD =  University of California San Diego Moores Cancer Center, VAPAHCS = Veterans Affairs Health Palo Alto Health Care System; Billings = Billings Medical Center; NSCLC =  Non-small Cell Lung Cancer; AJCC = American Joint Committee on Cancer; 10 M WBC = 10 Million White Blood Cells. SUV = Standardized Uptake Value; PVC = Partial Volume Correction. DICOM = Digital Imaging and Communication in Medicine.

* Variables are shown as median with interquartile range (IQR) for continuous variables and number with percent (%) for categorical or ordinal variables.

† Significant differences (p-value <0.05) by center.

§ As measured on PET-VCAR, n = 62 (see methods).

¶ Range provided instead of IQR.

∧ n = 62.

≈ Clinically retrieved value was used for this calculation when extracted data (from PET-VCAR) was not available.

The median time from PET to HD-CTC draw was 1 day (IQR 0–14 days, ranging from 24 before PET to 84 days after) and a median of 1.23 mL of blood (IQR 0.95–1.55) comprised a test for HD-CTC analysis per sample. Forty-four patients (62%) had a biopsy of the primary tumor during the course of their work-up and 27 (38%) of these biopsies were prior to HD-CTC draw, but only 2 were within 7 days–and none were within 24 hours–of HD-CTC sampling. Samples were usually processed within one day of phlebotomy (range 17–43 hours). Localized NSCLC predominated at SUMC and the PAVAHCS, while Billings and UCSD enrolled more locally advanced and metastatic patients (Table 1).

Sixty-two raw image (DICOM) files were available for quantitative image analysis, and 40 of 71 tumors required PVC adjustment based on phantom studies. Inter-scan variation across these four centers was within reported estimates (Supplementary File 1, Figure S3) [26]. Twenty-four of 62 images (39%) required manual override for automated tumor segmentation, and this override threshold varied from 45% to 70% compared to the default of 50%. Eight images were manually segmented as we could not threshold them properly. Reported median primary tumor SUV_max_ was 7.2 (IQR 3.7–15.5) and agreed well with extracted values from DICOM files (Supplementary File 1, Table S2), while SUV_maxPVC_ was slightly higher than the uncorrected metric (8.8, IQR 4.5–16.8).

Four non-NSCLC, metastatic nodules had a range of 0.0–2.2 HD-CTCs/10 M WBC (0–1.9 HD-CTCs/mL), while we detected a similar range of 0.0–2.2 HD-CTCs/10 M WBC for four benign nodules (0–0.8 HD-CTCs/mL). We therefore chose a threshold of >2.2 HD-CTCs/10 M WBC (>1.9 HD-CTCs/mL) for further analysis. Of note, no CTC clusters were detected in either of these groups.

The range of HD-CTCs/10 M WBC for NSCLC patients detected across centers varied from 0 to 779 for all TNM stages and from 0 to 695 in the 43 patients with stage I disease, which was similar to enumerated HD-CTCs/mL (Table 1). Greater than 2.2 HD-CTCs/10 M WBC were detected in 45/71 (63%) patients for all TNM stages and 27/43 (63%) patients with stage I disease only. For enumerated HD-CTCs/mL, 61% (43/71) of all TNM stages and 60% (26/43) of stage I patients had more than 2 HD-CTCs/mL. For all TNM stages, 33/71 patients (46%, median = 5, IQR 2–6) and 21/43 patients with stage I disease (49%, median = 6, IQR 2–10) had at least one HD-CTC cluster detected.

Maximum tumor FDG uptake, both uncorrected (SUV_max_) and corrected for tumor size (SUV_maxPVC_), was significantly different among stage I-IV NSCLC (p-value = 0.004 and 0.03 respectively). This was also true for histologic type, with squamous histology having higher values than untyped non-small cell lung cancers or adenocarcinoma (p-value = 0.0008 and 0.002 for SUV_max_ and SUV_maxPVC_ respectively). In contrast to this, HD-CTC numbers–whether per 10 M WBC, mL or enumerated by cluster count–did not vary significantly by TNM stage grouping (p-value = 0.64, 0.60 and 0.78 respectively) or by histologic type (p-value = 0.97, 0.96 and 0.90 respectively).

**Figure 3:**
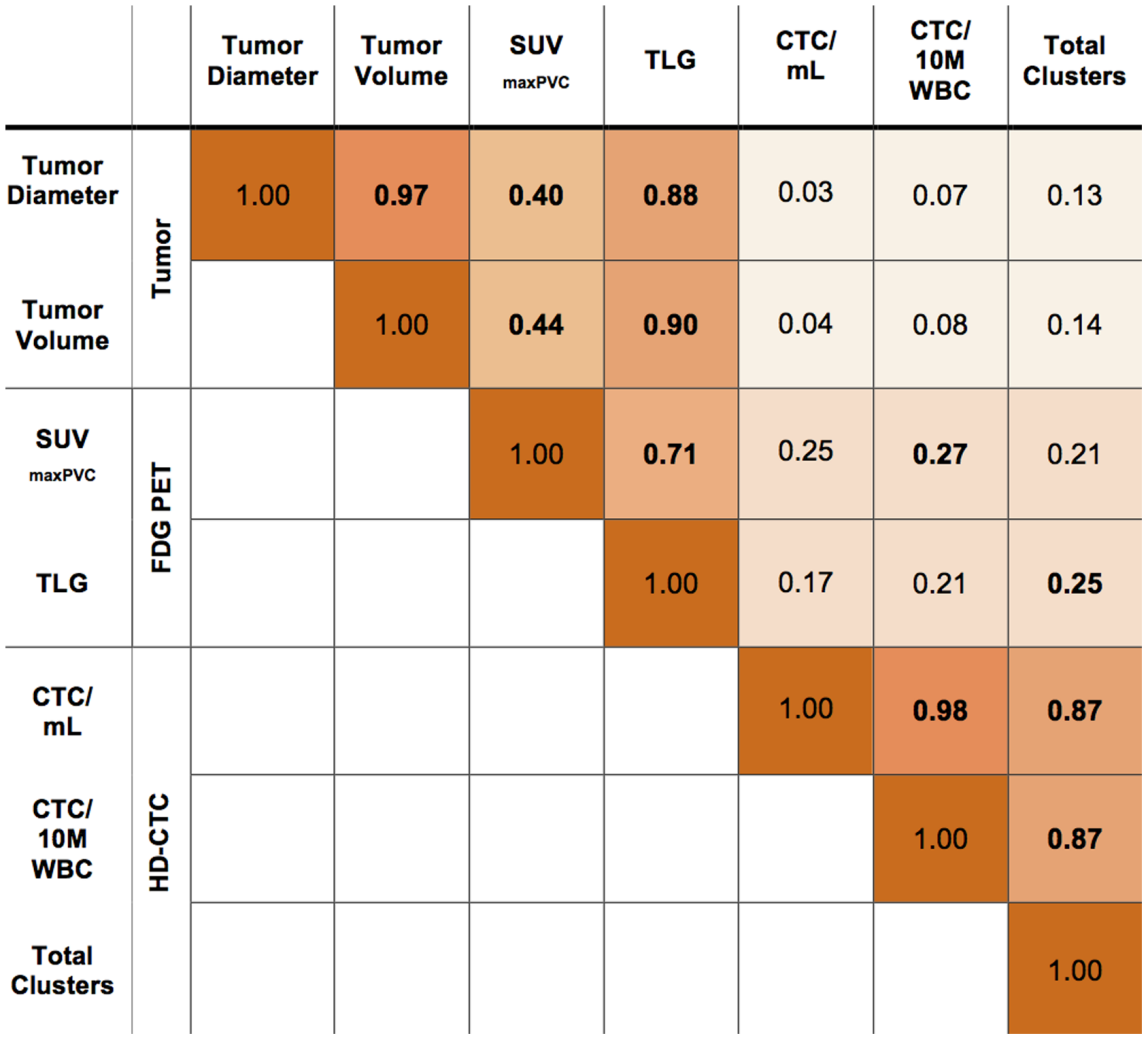
FDG Uptake and CTC Features Correlation Matrix*. TLG = Total Lesion Glycolysis; SUV = Standardized Uptake Value; PVC = Partial Volume Corrected; 10 M WBC = 10 Million White Blood Cells. Bolded numbers are significant by p-value <0.05. Half of the matrix only is presented since it is symmetric around one and correlations are shaded by the magnitude of correlation. *Spearman rank correlations are shown for 62 of 71 patients with data extracted by PET-VCAR.

HD-CTCs, per 10 M WBC, mL or by total clusters, did not correlate with tumor diameter as measured by CT (mm) or with extracted CT volume of the primary tumor (Figure 3). Increasing SUV_maxPVC_ correlated weakly with increasing HD-CTC counts and total clusters (Figure 3). When examining volume rather than scalar metrics, TLG correlated weakly with HD-CTC counts and total clusters when compared to CT volume, which did not correlate at all. These data were similar for log-normalized metrics (Supplementary File 1, Table S3). When the subgroup of patients who had imaging and phlebotomy within four weeks of each other only was analyzed (n = 65), correlations in Figure 3 were generally weaker than stronger.

**Figure 4:**
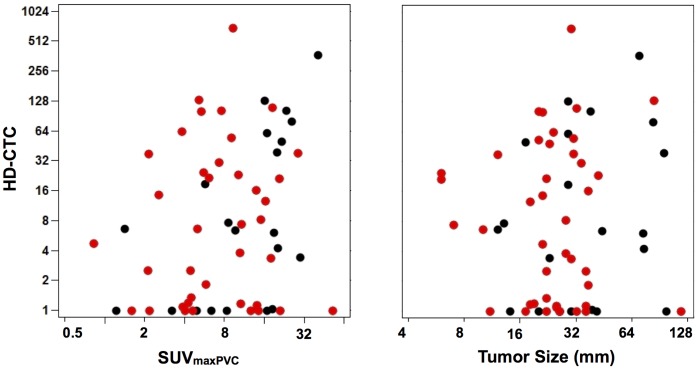
HD-CTC Scatter Plots for SUV_maxPVC_ and CT diameter*. Non-metastatic patients are highlighted in red (see methods for definition) and the axes are shown as log_2_(x,y) for ease of interpretation. Increasing SUV_maxPVC_ (left) was weakly correlated (r = 0.27, p-value = 0.03) with increasing HD-CTC/10 M WBC count compared to tumor diameter on CT (right; r = 0.07, p-value = 0.60), which showed no correlation. *Shown for 62 of 71 patients with data extracted by PET-VCAR.

We also plotted SUV_maxPVC_ by HD-CTC/10 M WBC to examine the structure of the data (Figure 4). Although these two variables were weakly correlated as described above, when examining SUV_maxPVC_ in the context of HD-CTC quantities, 8 PET “negative” tumors (SUV_maxPVC_ <2.5) had an HD-CTC/10 M WBC burden ranging from 0–38 while 19 PET “positive” tumors had no appreciable HD-CTC burden. Furthermore, for a given SUV_maxPVC_ or tumor diameter, there was a wide distribution of HD-CTCs in patient blood in both early and late stage NSCLC.

## Discussion

While multiple studies have examined the prevalence and prognostic utility of CTCs in carcinomas [27], including NSCLC [28], [29], only a few have assessed the relationship of individually enumerated CTCs with FDG PET-CT in the clinical setting [30]–[32]. These earlier studies focused on metastatic breast cancer [30], [31], but one recent study examined the change in CTC counts in response to treatment for relapsed lung cancer and this association with FDG PET SUV_max_ [32]. While this investigation was unable to find a predictive level for SUV_max_ and CTC response, the authors did note a trend for a change in CTC counts with treatment and initial FDG PET SUV_max_ of the relapsed tumor when stratifying by responders and non-responders. It is important to note that while this was a multi-center study, no FDG PET scanner calibration was performed.

To our knowledge, very few studies–if any–have aggregated a significant number of early-stage, treatment naïve patients with annotated imaging characteristics for NSCLC. Additionally, the above cited studies used EpCAM enriched cell-capture based platforms, which is an essential distinction since the non-EpCAM mediated approach we used here appears to be independent of TNM stage when compared to these other studies with poor yields for early-stage tumors [33]. While the HD-CTC assay has previously been applied NSCLC, [18], [19] this study extends the assay to a treatment-naïve setting with predominantly early-stage patients that are associated with imaging characteristics.

FDG uptake via SUV_max_ was strongly stage and histology dependent [15], [22] in this study, but the same associations were not evident for CTCs and we were unable to show even a modest correlation between these two biomarkers. This suggests that these two biomarkers may capture orthogonal snapshots of tumor biology, which together more aptly describe the biologic diversity of clinically similar NSCLC patients. As an example, we examined two stage IIIA (AJCC 7) patients who were treated the same (chemoradiation without surgery). Both patients had pre-treatment, glucoavid tumors (SUV_maxPVC_ 15.5 & 51.9), but discordant CTC counts (130 & 0). While the SUV_max_+/CTC+ patient has unfortunately expired at 9 months (with recurrence at 3 months), the SUV_max_+/CTC- patient remains disease free at nearly one year. This suggests that the T_4_N_0_ (NSCLC NOS) tumor without CTCs compared to the T_3_N_2_ (adenocarcinoma) tumor with nodal disease and many CTCs may be more accurately phenotyped with an integrated biomarker approach. Clearly though, this observation must be confirmed in more patients as we gather additional data over time.

The number of CTCs in the circulation is a function of primary tumor cell intravasation, tumor cell survival in the bloodstream, and tumor cell clearance from the bloodstream [11]. Previous data suggest that most CTCs are cleared from the circulation rapidly [34] and only a very small fraction proliferate at a distant site [35]. Our data showing that many CTCs exist in early-stage disease–either individually or in clusters–implies tumor bulk (i.e., more advanced disease) may not be the primary driver of the CTC steady state and CTC survival to metastasis. These findings are in agreement with one other previous study using non-EpCAM based CTC detection [29], need further confirmation in additional studies, and follow-up at the bench.

This study builds on previous work [21] examining the high prevalence of CTC clusters or tumor “microemboli” in advanced stage human carcinomas by showing that these deposits are numerous in early-stage disease as well. Since these “microemboli” have been reported to have a higher metastatic potential [36], CTC clusters could be important players in the biomarker milieu. A recent and provocative finding noted that CTC clusters display a primarily mesenchymal phenotype, which supports this hypothesis. [37] Yet, CTC aggregates appeared to correlate only weakly with FDG uptake of the primary tumor based on our pilot study.

We have established a clinical model to study lung cancer metabolism and its effect on patient outcome that may lead to additional and important observations, but this study has clear limitations that require discussion. Although we used a viable CTC detection platform, and we accounted for inter-scan and intra-scan variability by standardizing FDG PET-CT imaging across centers for treatment-naïve patients with varying tumor sizes, we were still left with a heterogeneous cohort of patients with respect to stage and histology–both of which can confound interpretation of FDG uptake and CTC analysis. We also captured samples that were drawn days to weeks from FDG PET-CT acquisition or near to a biopsy of the primary tumor. The effect of this variation may have introduced some error into our estimates of both CTC detection and FDG uptake, although the nature of this inaccuracy is not easy to estimate. Additionally, it is quite possible that analyzing epithelial characteristics of putative CTCs may not adequately define subpopulations with more mesenchymal or stem cell like properties [37] and that these subpopulations could associate differently with tumor glucose metabolism. Finally, we acknowledge that this is a pilot study and further patients are required to more fully study the utility of these data and to institute more sophisticated modeling given the non-linear distribution of the variables examined. Studying additional patients over time will build on these initial findings.

## Conclusion

We used a clinical model of non-small cell lung cancer that suggests while CTCs correlated weakly with tumoral FDG PET uptake, a large variation of CTC number for a given SUV_max_ or tumor diameter existed. We also noted that CTCs were prevalent in early-stage disease when using a non-enriched “fluid biopsy” CTC platform. These findings require further study and suggest that integrating complementary, non-invasive biomarkers may be useful for understanding patient heterogeneity in the early stages of this deadly disease.

## Supporting Information

File S1 Figure S1. Patient Flow Across Participating Medical Centers. Figure S2. Thoracic Phantom for FDG PET-CT Calibration. Figure S3. Recovery Coefficient (RC) Curves Across Centers. Table S1. Imaging Parameters for FDG PET-CT Acquisition by Center. Table S2. FDG PET-CT and Partial Volume Corrected Features by Patient. Table S3. Log-normalized Correlations.
(DOCX)
