# Supplementary material for: An Observational Study of Circulating Tumor Cells and 18F-FDG PET Uptake in Patients with Treatment-Naive Non-Small Cell Lung Cancer
Source: PLoS One. 2013 Jul 5;8(7):e67733. doi: 10.1371/journal.pone.0067733 (PMC3702496; doi:10.1371/journal.pone.0067733)
Supplement: File S1 — Figure S1. Patient Flow Across Participating Medical Centers. Figure S2. Thoracic Phantom for FDG PET-CT Calibration. Figure S3. Recovery Coefficient (RC) Curves Across Centers. Table S1. Imaging Parameters for FDG PET-CT Acquisition by Center. Table S2. FDG PET-CT and Partial Volume Corrected Features by Patient. Table S3. Log-normalized Correlations. (DOCX) [file pone.0067733.s001.docx]

**Supplementary Methods**

We used an Anthropomorphic Torso Phantom™ (Data Spectrum Corporation, Hillsborough, NC) to calibrate PET scanners across the four institutions in this study and for partial volume correction (Supplementary Figure 2). The phantom consisted of a mediastinal compartment (10.3 liters), two lung compartments filled with spherical styrofoam beads (0.8 liters each) and 10 hollow spheres ranging from 0.4 cm to 3.1 cm in diameter distributed within both lungs. Taken together, this phantom recapitulated the FDG activity of the mediastinal blood pool, background lung, and various tumor sizes with the lung. Each of these spheres was filled with a known and identical concentration of activity. At the time of imaging, the activity within the phantom was estimated at 39 MBq, the ratio of activity in the spheres to lungs was 10:1, and the ratio of activity for the spheres to the mediastinal compartment was 5:1. The phantom was scanned using each center’s current protocol for 2 beds position in list mode with CT attenuation and reconstruction parameters (Supplemental Table 1). The only parameter modified for the phantom study compared to clinical settings was the duration per bed position. For better statistical count values, the time was increased to 10 minutes per bed. Acquired imaging data was exported and tabulated for inter-scan calibration and partial volume correction by generating a recovery coefficient (RC) curve with % SUV_max_ scanned/SUV_max_ expected on the y-axis and tumor diameter on the x-axis (Supplementary Figure 3). The data were fit with a 4^th^ degree polynomial for extrapolation across all tumor diameters. For this curve, % predicted SUV_max_ (i.e, the recovery coefficient, RC) in all spheres was based on the 3.1 cm sphere, which was assumed to have no partial volume effect. We then calculated partial volume correction (PVC) according to the method of Vesselle[22] as follows: SUV_maxPVC_ = SUV_backgroundlung_ + ((SUV_max_ – SUV_backgroundlung_) / RC). Background lung FDG uptake values are provided in Supplementary Table 2 and were calculated from an adjacent region of interest to the primary tumor using a 2.0 cm three-dimensional ROI.

**Supplementary Figure 1**: Patient Flow Across Participating Medical Centers


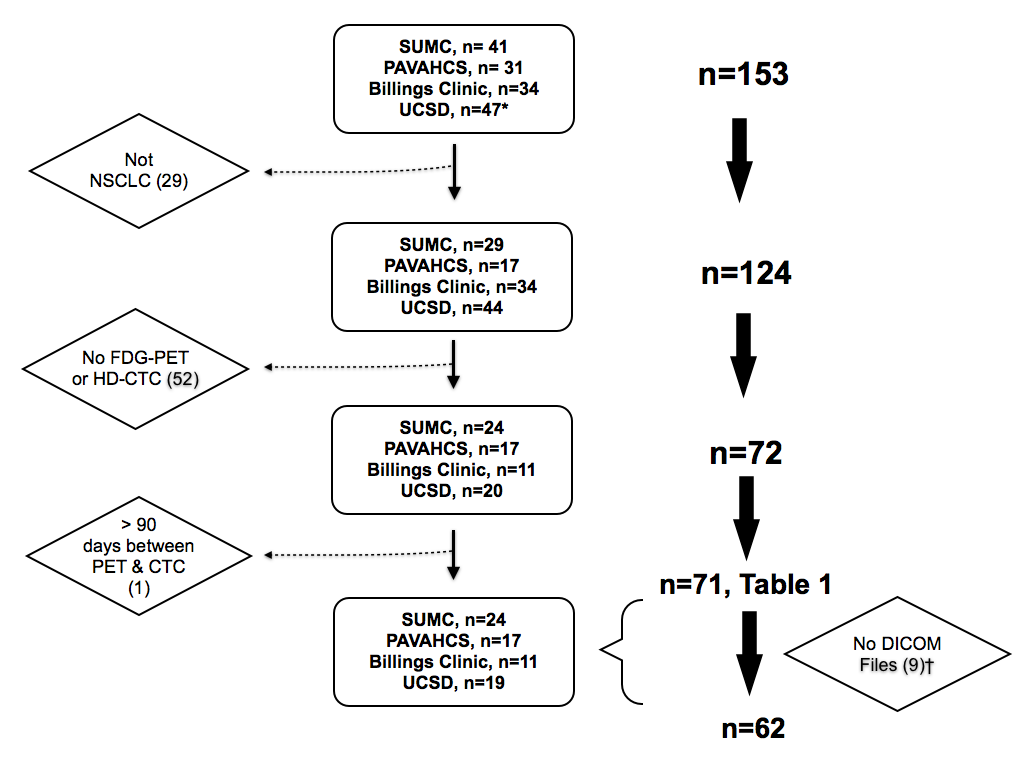


SUMC = Stanford University Medical Center; PAVAHCS = Palo Alto Veterans Affairs Healthcare System; UCSD = University of California San Diego Medical Center; DICOM = Digital Imaging Communications in Medicine. *Stage IV patients were not reviewed for eligibility. †Raw data was not available for these patients for further quantitative feature analysis.

**Supplementary Figure 2:** Thoracic Phantom for FDG PET-CT Calibration


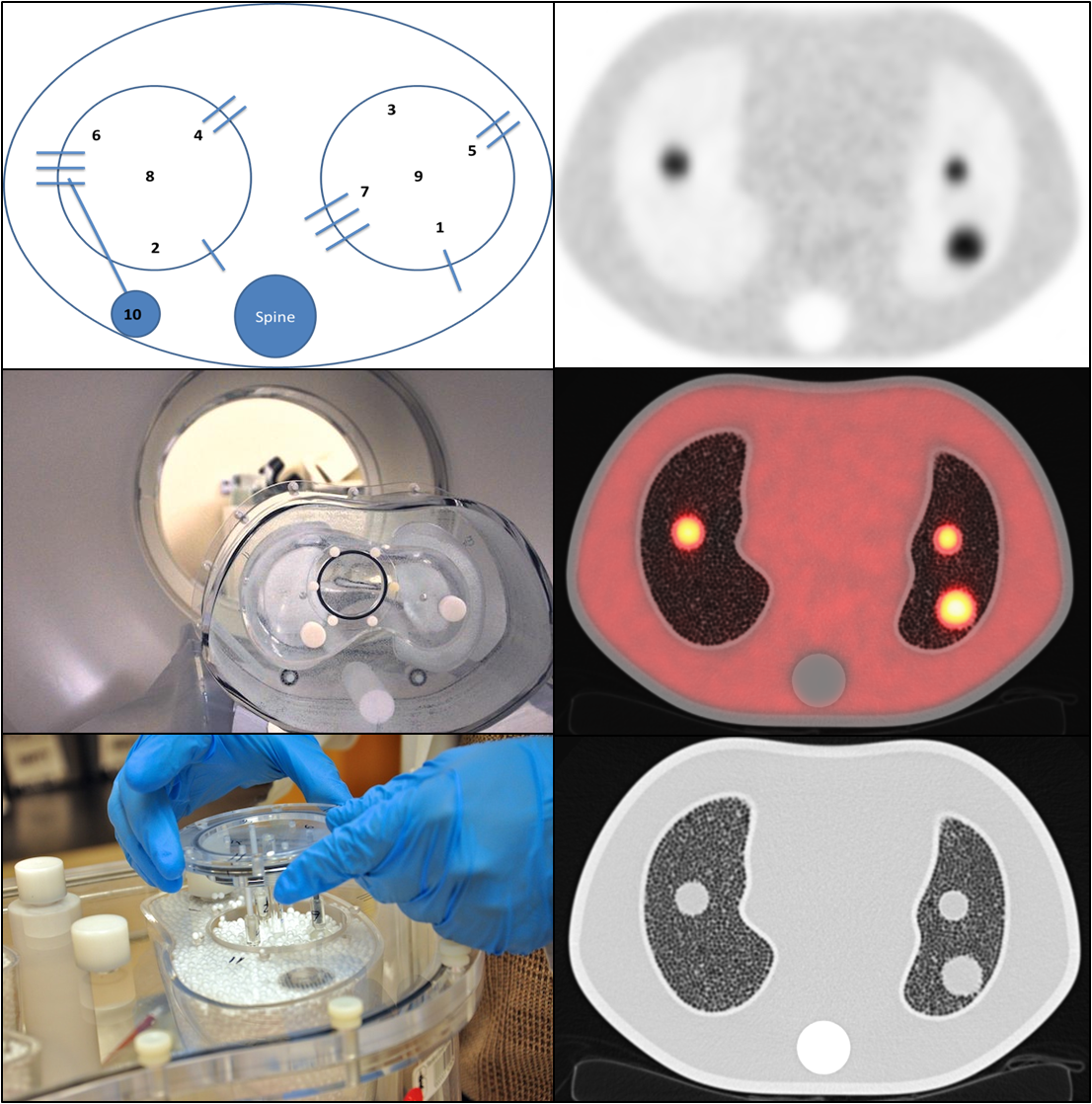


Schematics for the thoracic phantom (upper left), bed load and phantom assembly (middle and lower left) as well as post-imaging attenuation-corrected FDG PET, fusion of PET/CT, and CT images (upper, middle and lower right).

**Supplementary Figure 3:** Recovery Coefficient (RC) Curves Across Centers


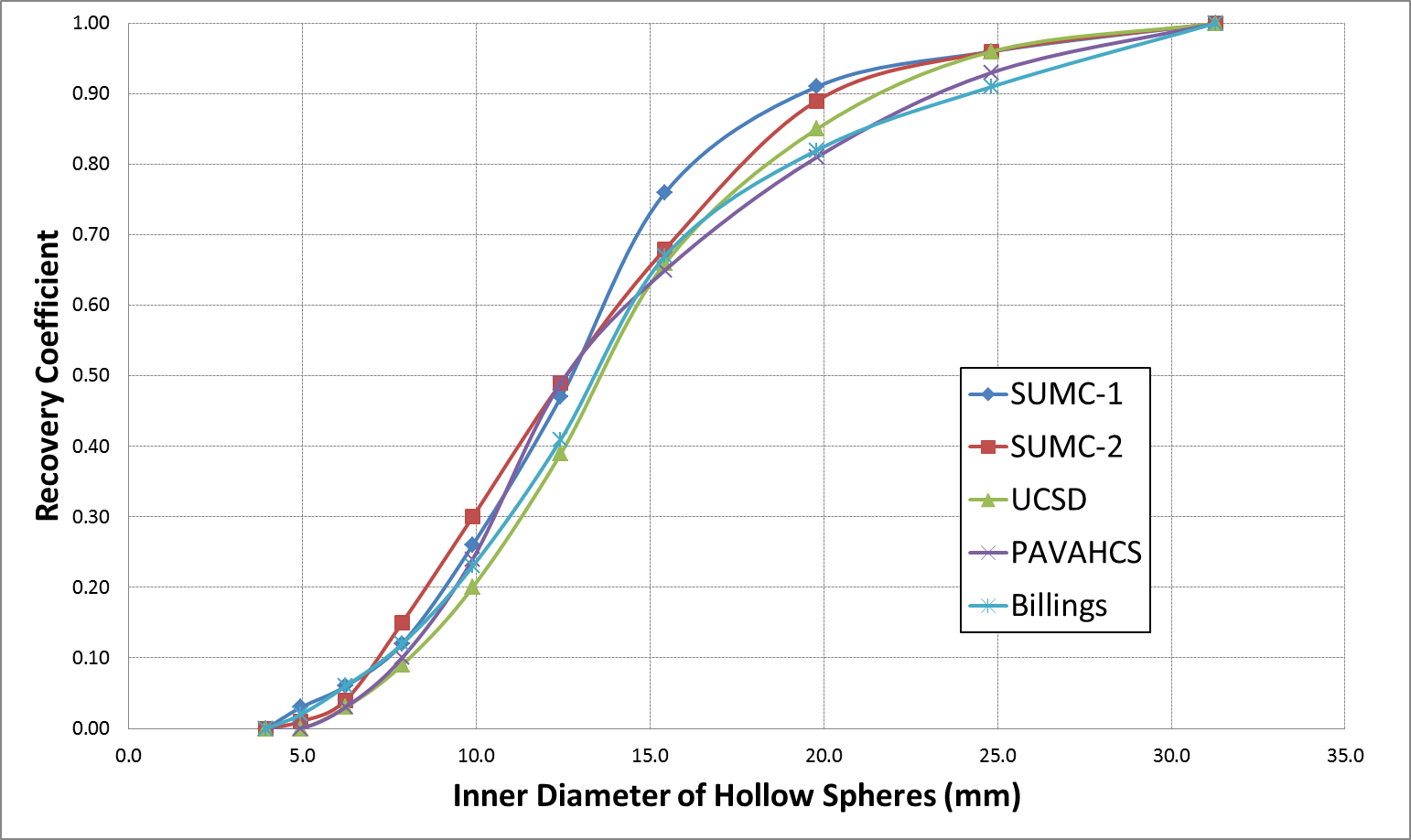


SUMC = Stanford University Medical Center; UCSD = University of California San Diego Medical Center; PAVAHCS = Palo Alto Veterans Affairs Health Care System.

**Supplementary Table 1:** Imaging Parameters for FDG PET-CT Acquisition by Center

| **Center** | **SUMC 1** | **SUMC 2** | **PAVAHCS** | **UCSD** | **BIllings** |  |
| --- | --- | --- | --- | --- | --- | --- |
| *Company* | GE | GE | GE | GE | Siemens | |
| *Model* | Discovery 690 | Discovery 600 | Discovery VCT | Discovery VCT | Biograph TruePoint | |
| *PET crystal* | LYSO | BGO | BGO | BGO | LSO | |
| *CT # slices* | 64 | 16 | 64 | 64 | 64 | |
| *Scout kVp* | 120 | 120 | 120 | 120 | 120 | |
| *Scout mA* | 10 | 10 | 10 | 10 | 35 | |
| *CT mode* | Helical & Full | Helical & Full | Helical & Full | Helical & Full | Helical & Full | |
| *Rotation time (s)* | 0.5 | 0.5 | 0.5 | 0.5 | 0.5 | |
| *Slice thickness (mm)* | 3.75 | 3.75 | 3.75 | 3.75 | 5.00 | |
| *CT kVp* | 140 | 140 | 120 | 140 | 120 | |
| *CT mA* | 85 | 75 | 100 | 100 | 340 | |
| *PET mode* | 3D | 3D | 3D | 3D | 3D | |
| *Bed number* | 2 | 2 | 2 | 2 | 2 | |
| *Time per bed (min)* | 10 | 10 | 10 | 10 | 10 | |
| *Matrix size* | 192 x 192 | 192 x 192 | 128 x 128 | 128 x 128 | 168 x 168 | |
| *Iteration* | 2 | 2 | 2 | 2 | 4 | |
| *Subsets* | 24 | 32 | 28 | 20 | 8 | |
| *Filter cut-off (mm)* | 6.4 | 6.4 | 6.0 | 6.4 | 6.0 | |

SUMC = Stanford University Medical Center; PAVAHCS = Palo Alto Veterans Affairs Health Care System; UCSD = University of California, San Diego Medical Center; kVp = kilovoltage potential; mA = milliamperage.

**Supplementary Table 2:** FDG PET-CT and Partial Volume Corrected Features by Patient*

| Center | Raw Data | PET Date | Dose (MBq) | TIme To Scan (minutes) | Glucose (mg/dL) | CT Volume | CT Diameter (mm) | RC (0-1) | PET Threshold (%) | SUV_max_ | SUV_max_  _PVC_ | TLG | SUV  _max_  _lung_† | SUV  _max_  _liver_¶ |
| --- | --- | --- | --- | --- | --- | --- | --- | --- | --- | --- | --- | --- | --- | --- |
| **Billings** | no | 9/16/10 | - | - | 103 | - | - | - | - | - | - | - | - | - |
| **Billings** | yes | 3/29/11 | 644 | 83 | 104 | 9.6 | 37 | 1.00 | 65 | 5.8 | 5.8 | 26.4 | 0.8 | 2.8 |
| **Billings** | yes | 4/14/11 | 570 | 94 | 110 | 10.4 | 29 | 0.97 | 50 | 16.2 | 16.7 | 95.5 | 0.7 | 2.8 |
| **Billings** | yes | 6/28/11 | 518 | 104 | 91 | 19.5 | 32 | 1.00 | 50 | 18.4 | 18.4 | 128 | 0.9 | 2.8 |
| **Billings** | yes | 12/27/11 | 537 | 79 | 104 | 4.6 | 18 | 0.77 | 60 | 12.7 | 16.3 | 28.9 | 0.8 | 4.0 |
| **Billings** | yes | 1/12/12 | 529 | 92 | 84 | 98.0 | 70 | 1.00 | 50 | 40.6 | 40.6 | 1061 | 0.6 | 3.9 |
| **Billings** | yes | 1/12/12 | 544 | 97 | 96 | 11.0 | 29 | 0.97 | 60 | 15.5 | 16.0 | 105 | 0.4 | 3.3 |
| **Billings** | yes | 12/29/12 | 500 | 77 | 120 | 18.0 | 38 | 1.00 | 50 | 23.4 | 23.4 | 215 | 0.4 | 3.3 |
| **Billings** | yes | 3/8/12 | 525 | 76.0 | 100 | 2.4 | 20 | 0.82 | 60 | 6.9 | 8.3 | 7.1 | 0.4 | 2.6 |
| **Billings** | yes | 3/5/12 | 537 | 89.0 | 118 | 145 | 83 | 1.00 | 50 | 25.5 | 25.5 | 1426 | 1.3 | 2.9 |
| **Billings** | yes | 4/16/12 | 570 | 88.0 | 99 | 483 | 117 | 1.00 | 50 | 51.9 | 51.9 | 10,366 | 0.6 | 3.1 |
| **UCSD** | no | 10/5/09 | 625 | 60? | 87 | - | - | - | - | 7.5 | - | - | 0.7 | 2.1 |
| **UCSD** | no | - | 429 | 60? | 91 | - | - | - | - | - | - | - | - | - |
| **UCSD** | no | - | 703 | 60? | 99 | - | - | - | - | - | - | - | - | - |
| **UCSD** | no | 11/17/09 | 574 | 60? | 94 | - | - | - | - | 19.1 | - | - | 1.0 | 3.6 |
| **UCSD** | no | - | 699 | 60? | 88 | - | - | - | - | - | - | - | - | - |
| **UCSD** | yes | 1/15/10 | 514 | 50.0 | 114 | 406 | 95 | 1.00 | 50 | 19.8 | 19.8 | 2390 | 0.6 | 1.9 |
| **UCSD** | no | - |  | 60? | - | - | - | - | - | - | - | - | - | - |
| **UCSD** | yes | 4/26/10 | 662 | 85.0 | - | 196 | 73 | 1.00 | 50 | 18.9 | 18.9 | 1290 | 0.3 | 2.2 |
| **UCSD** | yes | 10/26/10 | 640 | 88.0 | 97 | 6.4 | 23 | 0.90 | 70 | 26.8 | 29.7 | 83.7 | 0.5 | 2.6 |
| **UCSD** | no | 5/23/11 | 592 | 60? | 110 | - | - | - | - | 9.2 | - | - | 0.8 | 3.1 |
| **UCSD** | yes | 6/1/11 | 677 | 58.0 | - | 22.3 | 37 | 1.00 | 50 | 13.8 | 13.8 | 93.1 | 0.8 | 2.7 |
| **UCSD** | yes | 9/28/11 | 766 | 68.0 | 284 | 87 | 84 | 1.00 | 50 | 5.1 | 5.1 | 218 | 0.7 | 1.8 |
| **UCSD** | yes | 12/9/11 | 696 | 90.0 | 96 | 1.7 | 17 | 0.72 | - | 1.4 | 1.6 | 2.5 | 0.9 | 2.4 |
| **UCSD** | no | 1/9/12 | 611 | 60? | 113 | - | - | - | - | 22.7 | - | - | 0.4 | 2.0 |
| **UCSD** | yes | 1/24/12 | 588 | 55.0 | 81 | 11.3 | 30 | 0.99 | 50 | 17.7 | 17.9 | 77.3 | 0.6 | 3.4 |
| **UCSD** | yes | 2/10/12 | 751 | 78.0 | 104 | 20.3 | 36 | 1.00 | 60 | 2.1 | 2.1 | 18.5 | 0.8 | 2.8 |
| **UCSD** | yes | 1/18/12 | 518 | 65.0 | - | 5.4 | 28 | 0.97 | 50 | 14.5 | 14.9 | 33.4 | 0.7 | 3.0 |
| **UCSD** | yes | 5/9/12 | 681 | 61.0 | - | 146 | 98 | 1.00 | 60 | 6.3 | 6.3 | 147 | 0.6 | 2.0 |
| **UCSD** | yes | 5/10/12 | 747 | 58.0 | - | 14.8 | 41 | 1.00 | 70 | 3.2 | 3.2 | 27.5 | 0.7 | 2.3 |
| **PAVAHCS** | yes | 6/20/11 | 622 | 61 | 90 | 2.7 | 21 | 0.84 | 65 | 2.2 | 2.5 | 4.0 | 0.4 | 2.8 |
| **PAVAHCS** | yes | 6/23/11 | 588 | 51 | 98 | 2.6 | 19 | 0.78 | 50 | 3.4 | 4.2 | 3.5 | 0.5 | 2.4 |
| **PAVAHCS** | yes | 6/29/11 | 525 | 58 | 109 | 1.2 | 12 | 0.44 | - | 1.5 | 2.1 | 2.5 | 1.0 | 3.2 |
| **PAVAHCS** | yes | 6/29/11 | 599 | 49 | 97 | 4.0 | 22 | 0.87 | 65 | 3.9 | 4.4 | 7.9 | 0.5 | 3.0 |
| **PAVAHCS** | yes | 6/2/11 | 648 | 80 | 118 | 2.7 | 17 | 0.71 | 50 | 15.5 | 21.5 | 26.6 | 0.8 | 2.6 |
| **PAVAHCS** | yes | 7/13/11 | 511 | 69 | - | 30.9 | 39 | 1.00 | 50 | 18.3 | 18.3 | 381 | 0.7 | 2.3 |
| **PAVAHCS** | yes | 9/12/11 | 481 | 120 | 123 | 10.0 | 31 | 1.00 | 50 | 28.4 | 28.4 | 142 | 0.4 | 3.3 |
| **PAVAHCS** | yes | 11/16/11 | 599 | 59 | 151 | 0.9 | 11 | 0.34 | 70 | 1.7 | 4.0 | 1.0 | 0.5 | 2.4 |
| **PAVAHCS** | yes | 1/24/12 | 551 | 60 | 98 | 4.5 | 26 | 0.94 | 50 | 11.8 | 12.5 | 24.0 | 0.5 | 3.1 |
| **PAVAHCS** | yes | 1/30/12 | 562 | 56 | 99 | 8.7 | 29 | 0.98 | 50 | 6.6 | 6.7 | 23.6 | 0.7 | 2.5 |
| **PAVAHCS** | yes | 2/7/12 | 503 | 56 | - | 11.1 | 34 | 1.00 | 60 | 7.2 | 7.2 | 21.0 | 0.4 | 2.6 |
| **PAVAHCS** | yes | 2/14/12 | 625 | 96 | 96 | 2.7 | 22 | 0.87 | 60 | 4.0 | 4.5 | 9.4 | 0.9 | 3.2 |
| **PAVAHCS** | yes | 3/9/12 | 529 | 69 | - | 0.2 | 6 | 0.02 | - | 0.6 | 5.5 | 0.0 | 0.5 | 3.7 |
| **PAVAHCS** | yes | 4/6/12 | 555 | 65 | - | 0.9 | 14 | 0.58 | - | 1.0 | 1.2 | 1.4 | 0.7 | 2.5 |
| **PAVAHCS** | yes | 4/11/12 | 500 | 62 | - | 9.0 | 30 | 0.99 | 50 | 16.6 | 16.8 | 73.8 | 0.4 | 4.2 |
| **PAVAHCS** | yes | 4/20/12 | 492 | 73 | - | 6.8 | 29 | 0.98 | 50 | 5.6 | 5.7 | 21.0 | 0.9 | 2.7 |
| **PAVAHCS** | yes | 5/24/12 | 463 | 63 | - | 85.1 | 74 | 1.00 | 45 | 20.0 | 20.0 | 489 | 0.9 | 2.0 |
| **SUMC** | yes | 8/5/11 | 437 | 100 | 141 | 5.4 | 20 | 0.90 | 50 | 6.9 | 7.6 | 14.9 | 0.8 | 3.8 |
| **SUMC** | yes | 8/8/11 | 355 | 53 | 134 | 7.2 | 25 | 0.96 | 60 | 3.7 | 3.8 | 14.1 | 0.6 | 3.2 |
| **SUMC** | yes | 8/23/11 | 477 | 60 | 108 | 3.3 | 21 | 0.93 | - | 0.8 | 0.8 | 1.8 | 0.5 | 2.7 |
| **SUMC** | yes | 9/1/11 | 437 | 60 | 91 | 22.2 | 36 | 1.00 | 50 | 13.9 | 13.9 | 146 | 0.6 | 2.8 |
| **SUMC** | yes | 9/14/11 | 389 | 60 | 115 | 13.8 | 32 | 1.00 | 60 | 4.6 | 4.6 | 30.5 | 0.7 | 2.8 |
| **SUMC** | yes | 10/4/11 | 488 | - | 121 | 10.2 | 31 | 1.00 | 50 | 9.0 | 9.0 | 53.4 | 0.9 | 3.1 |
| **SUMC** | yes | 10/19/11 | 477 | 60 | 100 | 6.2 | 24 | 0.96 | 60 | 3.7 | 3.8 | 18.3 | 0.8 | 3.4 |
| **SUMC** | yes | 11/11/11 | 477 | 76 | 100 | 8.1 | 28 | 0.99 | 50 | 10.3 | 10.4 | 39.6 | 0.9 | 3.0 |
| **SUMC** | yes | 11/11/11 | 414 | 75 | 93 | 8.2 | 26 | 0.97 | 60 | 4.8 | 4.9 | 16.3 | 0.5 | 2.7 |
| **SUMC** | yes | 11/16/11 | 451 | 72 | 117 | 6.0 | 22 | 0.90 | 50 | 19.0 | 21.0 | 65.6 | 0.9 | 2.3 |
| **SUMC** | yes | 11/7/11 | 551 | 60 | 104 | 14.1 | 36 | 1.00 | 50 | 14.4 | 14.4 | 71.4 | 1.1 | 4.9 |
| **SUMC** | yes | 12/7/11 | 426 | 53 | 79 | 23.4 | 42 | 1.00 | 50 | 10.1 | 10.1 | 164 | 0.9 | 3.7 |
| **SUMC** | yes | 12/12/11 | 488 | 88 | 108 | 13.6 | 44 | 1.00 | 50 | 9.6 | 9.6 | 74.1 | 0.9 | 2.1 |
| **SUMC** | yes | 12/20/11 | 400 | 55 | 112 | 6.6 | 26 | 0.97 | 65 | 2.2 | 2.2 | 7.9 | 0.7 | 2.5 |
| **SUMC** | yes | 12/29/11 | 477 | 59 | 103 | 2.0 | 18 | 0.80 | 60 | 8.5 | 10.5 | 8.0 | 0.4 | 1.9 |
| **SUMC** | yes | 1/23/12 | 400 | 45 | 99 | 1.7 | 13 | 0.53 | 50 | 4.8 | 8.5 | 6.1 | 0.7 | 2.0 |
| **SUMC** | yes | 2/7/12 | 463 | 49 | 101 | 5.0 | 30 | 0.99 | 60 | 9.1 | 9.2 | 34.9 | 0.5 | 2.5 |
| **SUMC** | yes | 2/13/12 | 385 | 62 | 89 | 0.9 | 12 | 0.44 | - | 1.0 | 1.4 | 0.6 | 0.8 | 2.8 |
| **SUMC** | yes | 3/1/12 | 455 | 60 | 108 | 0.2 | 7 | 0.04 | 50 | 1.1 | 10.7 | 0.7 | 0.7 | 3.5 |
| **SUMC** | yes | 3/7/12 | 463 | 70 | 105 | 3.7 | 21 | 1.00 | 70 | 5.3 | 5.3 | 8.5 | 0.6 | 3.1 |
| **SUMC** | yes | 3/9/12 | 466 | 60 | 71 | 0.7 | 10 | 0.25 | - | 1.7 | 5.0 | 1.0 | 0.6 | 3.3 |
| **SUMC** | yes | 3/9/12 | 411 | 60 | 96 | 5.9 | 22 | 0.90 | 60 | 5.6 | 6.1 | 19.4 | 0.9 | 3.2 |
| **SUMC** | yes | 3/13/12 | 374 | 45 | 112 | 0.2 | 6 | 0.02 | - | 1.1 | 20.7 | 0.7 | 0.7 | 3.0 |
| **SUMC** | yes | 3/2/12 | 559 | 60 | 61 | 8.0 | 26 | 0.95 | 65 | 2.1 | 2.2 | 11.0 | 0.7 | 2.6 |

SUMC = Stanford University Medical Center; UCSD= University of California San Diego Medical Center; VAPAHCS = Veterans Affairs Palo Alto Health Care System; RC = Recovery Coefficient; TLG = Total Lesion Glycolysis. *Quantitative PET and CT features were extracted from the PET-VCAR implementation for 62 patients with DICOM files (see Methods). †See Supplementary Methods. ¶Using an approximate 2 cm region of interest (ROI) over the right hepatic lobe.

**Supplementary Table 3:** Log-normalized Correlations*

| Variable | Test | Tumor  Diameter | Tumor  Volume | SUV  max  PVC | TLG |
| --- | --- | --- | --- | --- | --- |
| HD-CTC/10M WBC | Spearman rank | 0.068 | 0.082 | **0.275** | 0.207 |
|  | Kendall’s Tau | 0.043 | 0.059 | **0.189** | 0.141 |
| Total clusters | Spearman rank | 0.131 | 0.137 | 0.215 | **0.253** |
|  | Kendall’s Tau | 0.102 | 0.108 | 0.165 | **0.190** |

PVC = Partial Volume Corrected; TLG = Total Lesion Glycolysis. All variables were log-normalized for this analysis. Variables with zero values were continuity corrected by adding one to all values. *Bold values are significant at the p-value < 0.05 level.
